# Comparative genomic analysis of *Paenibacillus* sp. SSG-1 and its closely related strains reveals the effect of glycometabolism on environmental adaptation

**DOI:** 10.1038/s41598-017-06160-9

**Published:** 2017-07-18

**Authors:** Hui Xu, Shishang Qin, Yanhong Lan, Mengjia Liu, Xiyue Cao, Dairong Qiao, Yu Cao, Yi Cao

**Affiliations:** 1 Microbiology and Metabolic Engineering of Key Laboratory of Sichuan Province, College of Life Science, Sichuan University, Chengdu, 610065 P.R. China; 2 College of Food Science, Northeast Agricultural University, Harbin, 150030 P.R. China

## Abstract

The extensive environmental adaptability of the genus *Paenibacillus* is related to the enormous diversity of its gene repertoires. *Paenibacillus* sp. SSG-1 has previously been reported, and its agar-degradation trait has attracted our attention. Here, the genome sequence of *Paenibacillus* sp. SSG-1, together with 76 previously sequenced strains, was comparatively studied. The results show that the pan-genome of *Paenibacillus* is open and indicate that the current taxonomy of this genus is incorrect. The incessant flux of gene repertoires resulting from the processes of gain and loss largely contributed to the difference in genomic content and genome size in *Paenibacillus*. Furthermore, a large number of genes gained are associated with carbohydrate transport and metabolism. It indicates that the evolution of glycometabolism is a key factor for the environmental adaptability of *Paenibacillus* species. Interestingly, through horizontal gene transfer, *Paenibacillus* sp. SSG-1 acquired an approximately 150 kb DNA fragment and shows an agar-degrading characteristic distinct from most other non-marine bacteria. This region may be transported in bacteria as a complete unit responsible for agar degradation. Taken together, these results provide insights into the evolutionary pattern of *Paenibacillus* and have implications for studies on the taxonomy and functional genomics of this genus.

## Introduction

The genus *Paenibacillus* was designated in 1993 and was then composed of 11 species originally belonging to the genus *Bacillus* ^1^. Novel species of this genus have been rapidly discovered, and currently, more than 150 named species have been identified. Members of this genus are facultative anaerobic, endospore-forming, motile and rod-shaped bacteria. Most of these bacteria are Gram positive, while others are Gram negative or variable^2–4^. The genus *Paenibacillus* is physiologically, biochemically and morphologically diverse and is present in various environments, such as soil^5^, spring water^6^, insect larvae^7^ and human feces^4^. Corresponding to its adaptability to a wide range of environment, the specific biological characteristics of these bacteria vary. This group initially received attention because of the excellent ability to promote plant growth in some species, such as *Paenibacillus polymyxa*, via several mechanisms, including phosphate solubilisation and nitrogen fixation^8^. *Paenibacillus larvae* has been reported as one of two bacterial species pathogenic for American Foulbrood (AFB), a fatal, globally spread epizootic disease^7^. These bacteria have also been noted for their ability to hydrolyze a variety of carbohydrates, including cellulose, starch, and xylan^9, 10^. Whole-genome sequencing has provided insights into the molecular mechanisms of these unique attributes. Djukic *et al*. identified toxic proteins of *Paenibacillus larvae* based on genome searching and compared the pathogenic mechanisms of two genotypes, ERIC I and ERIC II^11^. Dsouza *et al*. comparatively analyzed Antarctic and temperate species of *Paenibacillus* and identified traits of *Paenibacillus darwinianus* that enable it to withstand extremely cold environments^12^. Recently, Xie *et al*., using 35 newly sequenced or previously reported *Paenibacillus* genomes, systematically studied the mechanisms of plant growth promotion^13^. However, the reason these strains exhibit broad environmental adaptability remains unknown.

Comparative genomics revealed that the diversity of the gene repertoires endows these microorganisms with various metabolic activities and extensive adaptabilities to the environment^14–18^. In this context, the key unit of microbial evolution is not the genome of an individual bacterium but, rather, the pan-genome of a prokaryote species. Moreover, comparative genomics indicated that the gene repertoires of species are dynamic, and the incessant flux reflects expansion through horizontal gene transfer (HGT), gene duplication, the potential *de novo* emergence of genes, and contraction via gene loss and genome reduction^19^. Many obligate endosymbionts of insects lack genes considered essential in other bacteria, and the genome size of some bacterial symbionts is even reduced to 150 kb^20^. By contrast, some prokaryotic organisms have enormous gene sets of as many as 13 Mb for responding to complex environments^21, 22^. Bacteria of the *Paenibacillus* genus show the extensive environmental adaptability and the broad range of genome size^23, 24^. However, research on adaptability of this genus based on genome dynamics is not available. To date, more than 100 *Paenibacillus* strains, covering a variety of habitats, have been sequenced, and these large available genome data make it possible for a comprehensive understanding of the adaptability of these microbes to environment.

Additionally, our lab has isolated the soil bacterium *Paenibacillus* sp. SSG-1, which exhibits agar degradation^25^. We comprehensively examined the characters of *Aga1*, an agarase gene in *Paenibacillus* sp. SSG-1, and proposed that this gene was acquired through HGT^26^. However, we subsequently found that the HGT event was involved in not only *Aga1* gene but also a large region. In the present study, we sequenced the genome of *Paenibacillus* sp. SSG-1, conducted a comparative genomic analysis of SSG-1 using 76 previously sequenced strains (75 *Paenibacillus spp*. and 1 *bacillus subtilis*), and constructed a pan-genome for *Paenibacillus*. The 75 selected *Paenibacillus spp*. were originated from various niches, such as diverse soil, water, plant and human isolates, and their genomes are available in the public database. The present study examined the effects of genome dynamics on the adaptability of *Paenibacillus* species to disclose the evolution pattern of the genus *Paenibacillus*. Moreover, the HGT event endowing *Paenibacillus* sp. SSG-1 with the agar-degrading trait was further investigated.

## Results

### Genome features of *Paenibacillus* sp. SSG-1

**Figure 1:**
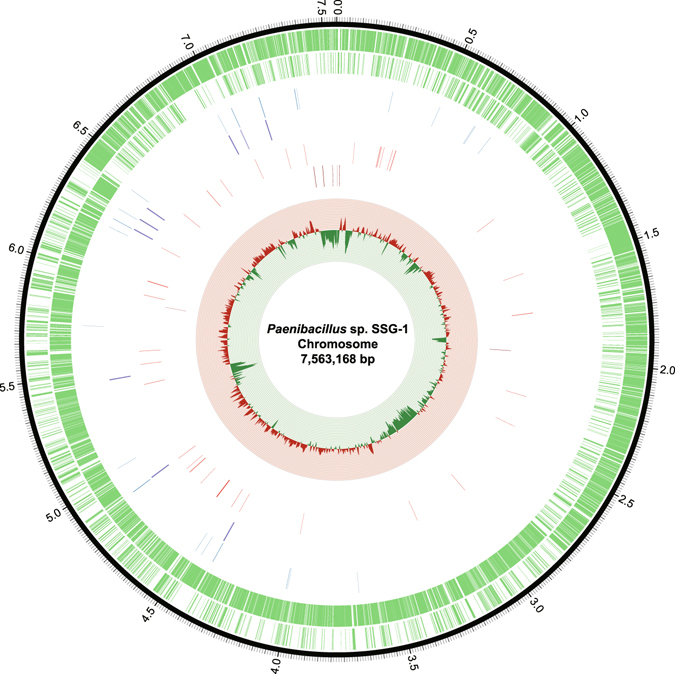
Circular diagrams of the *Paenibacillus* sp. SSG-1 chromosome. Information from the outermost circle to the innermost circle provide the following data: (1) position in megabases (black); (2) forward strand CDSs (green); (3) reverse strand CDSs (green); (4) tRNAs (blue); (5) rRNAs (purple); (6) repeats (red); (7) ISs and TEs (dark red); and (8) deviation of the GC content per 5000 bp compared with the global genome (positive: red; negative: green; wave range: −0.1714~0.0832).

The genome of *Paenibacillus* sp. SSG-1 was sequenced. The genomic features of SSG-1 are shown in Fig. 1 and Table 1, and those of other genus members are shown in Dataset S1. The SSG-1 genome comprises a 7.56 Mb chromosome, which is larger than most other members of the genus (4.05~8.82 Mb, mean 6.50 Mb). A similar result was observed in protein-coding gene number, for which SSG-1 has 6,659 genes and other strains have 3,668 to 7,522 genes (mean 5,476). The GC content of SSG-1 is 0.5305, while that of other strains is 0.4134 to 0.6327 (mean 0.4879). The result from scanning the SSG-1 genome shows that the GC content is unevenly distributed and undergoes relatively dramatic changes in some location, and that abundant insert sequences (IS) and transposon sequences (TE) are present in SSG-1 genome. It suggests that a large number of HGT events occurred in the evolution of SSG-1.

**Table 1:** General features of *Paenibacillus* sp. SSG-1 genome.

Category	Number
Genome size (nt)	7,563,168
G + C content (%)	0.5305
Protein-coding gene	6,812
Genes with assigned function	5,827
tRNA	87
rRNA	25
sRNA	48
Tandem repeat	231
Transposon	20
IS element	17

### Pan-genome analysis reveals great difference in *Paenibacillus*

In order to ensure the reliability of subsequent analysis, we implemented a filtering process for all downloaded gene sets (see section “Materials and Methods” for more information) and performed BUSCO assessment^27^ of filtered gene sets (together with *Paenibacillus* sp. SSG-1). The average of completeness of the gene sets is 96.5% (based on bacillales_odb9 lineage dataset) (Table S1), which indicates that the datasets are highly reliable and suitable for the subsequent analyses.

**Figure 2:**
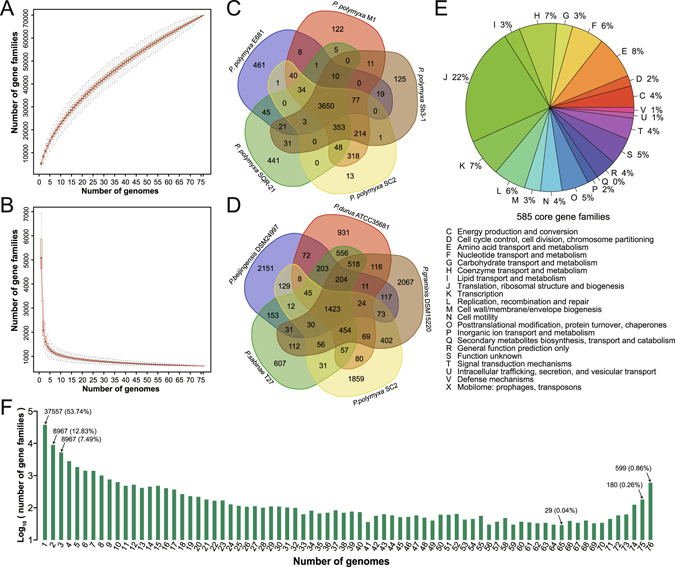
Pan-genome analysis of *Paenibacillus*. (**A**) The pan-genome accumulation curve. (**B**) The core-genome accumulation curve. (**C**) Venn diagram of the gene families in five *Paenibacillus polymyxa* strains. (**D**) Venn diagram of the gene families in five different species of *Paenibacillus*. (**E**) Functional distribution of the gene families in the core-genome. (**F**) Distribution of gene families shared in different numbers of genomes.

Using the reciprocal best hit method^28^, the homology relationship of genes of species were constructed. Among 76 *Paenibacillus* spp., 416,204 coding genes were clustered in 69,882 gene families. The number of gene families in strains ranges from 3,288 to 6,934 (mean 5,244), which largely consistent with the number of genes, indicating that most genes do not have multiple copies. The number of common gene family is 599, and genes clustered in common gene families occupies 8.64% to 18.22% (mean 11.69%) of total genes in each strain (Figure S1). The number of strain unique gene families ranges from 9 to 1469 in each strain, and genes clustered in unique gene families occupies 0.18% to 29.82% (mean 9.26%). Among 69,882 gene families, only 599 (0.86%) are shared by all 76 *Paenibacillus* spp., but up to 37,557 (53.74%) are uniquely found in one strain (Fig. 2F), indicating that there is the great difference in these genomes. In more detail, we selected five high-quality genomes of *Paenibacillus polymyxa* to investigate intra-species difference. Total 6,052 gene families were identified, and 3,650 (60.31%) are shared by the five strains (Fig. 2C). Although the five strains are the same species, about 20% of the gene families in their genomes are not conserved. Similarly, we chose five high-quality genomes of different species to investigate inter-species difference. The result shows the difference among the five strains greatly increased. Total 12,601 gene families were identified, but only a very small part (1,423, 10.99%) is conserved (Fig. 2D). All of the above results indicate that the difference in the genomes of *Paenibacillus* is very large. Unlike *Nocardiopsis* spp. which showed the largest proportion of genes are conserved (43.1%)^17^, *Paenibacillus* spp. have only a small portion of the conserved genes and more genes are varied.

Furthermore, to estimate the size of the pan-genome for the genus *Paenibacillus*, the number of gene families after continually adding new genome data was analyzed during 1,000 random duplicate tests. The size of the pan-genome rapidly increased with each addition (mean 862 for each increase), even when the final genome was added (Fig. 2A). Similarly, to estimate the size of the core-genome for the genus *Paenibacillus*, the number of shared gene families after continually adding new genome data was analyzed during 1,000 random duplicate tests. The size of core-genome initially sharply descended and finally relatively stabilized at a minimum of 599 gene families (Fig. 2B). A great substantial change was found in the size of the pan-genome after the addition of new genomes, indicating that the pan-genome of *Paenibacillus* is open. The open pan-genome suggests that *Paenibacillus* tend to change genomic content to adapt to the environment.

Core-genome is expected to be important or essential in all *Paenibacillus*. Thus, we used Cluster of Orthologous Groups (COG) assignment to categorize the functions of gene families in *Paenibacillus* core-genome (Fig. 2E). The result shows that genes involved in translation, ribosomal structure and biogenesis (Category J) occupies largest portion in core genome, indicating that most of these genes are housekeeping. We used *Paenibacillus* sp. SSG-1 as the reference and performed GO enrichment analysis of its core genes. Besides housekeeping functions, the significant functions include flagellum assembly (GO:0044780), flagellum-dependent cell motility (GO:0071973) and chemotaxis (GO:0006935) (Table S2). It suggests that mobility is important to *Paenibacillus*. In complex environments, *Paenibacillus* could move towards regions where there are higher concentrations of nutrients or other beneficial conditions for survival. With respect to the entire pan-genome of 76 *Paenibacillus* spp., carbohydrate transport and metabolism (Category G) and transcription (Category K) are most abundant in the COG assigned gene families (Figure S2A). A similar result was obtained from functional distribution of individual gene set (Figure S2B). These results show that a large proportion of expanded genes in genomes of *Paenibacillus* is responsible for glycometabolism and transcriptional regulation.

### Phylogenetic analysis contributes to taxonomy of *Paenibacillus*

As a molecular marker, 16S rDNA is widely used for strain identification, but the sensitivity of this method for sub-genus distinction is markedly decreased. Because the 16S rDNA sequences of *Paenibacillus* species show high similarity, the phylogenetic tree based on these sequences is ambiguous with a low value of bootstraps in some nodes (Figure S3). The phylogeny based on the core-genome remarkably overcomes this problem and has become a standard in the last few years.

**Figure 3:**
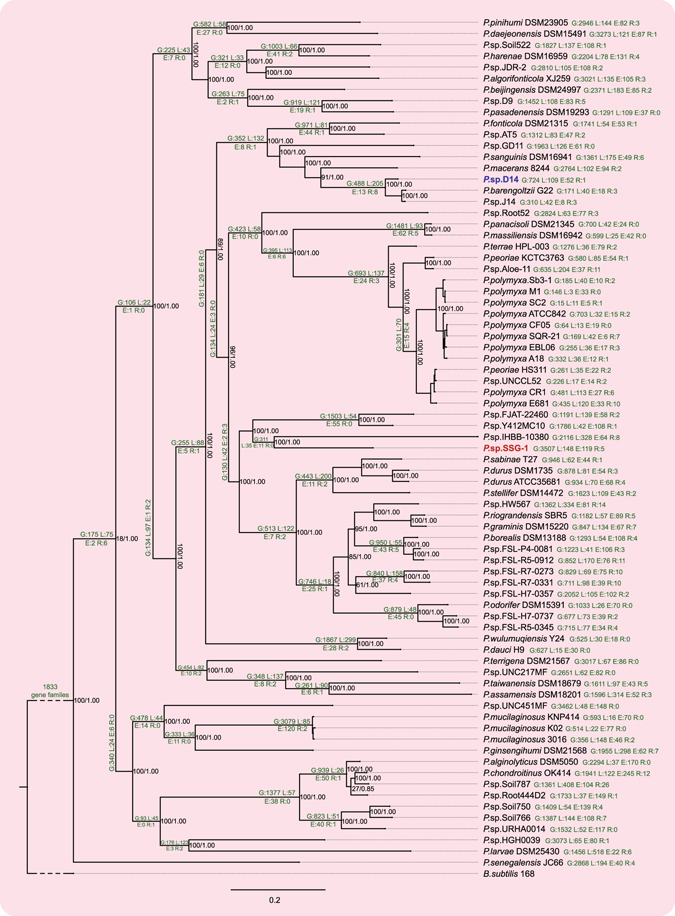
Phylogenetic tree of *Paenibacillus*. The numbers in nodes denote the bootstrap value (maximum 100) and Bayesian posterior probability (maximum 1.00). The numbers in branches denote the numbers of four categories of the change events (G: gain; L; loss; E: expansion; R: reduction). Numbers in short branches or in crowded nodes are omitted.

In the present study, the core-genome phylogeny of the genus *Paenibacillus* was investigated. The *Bacillus subtilis* 168^29^ with latest genome was selected an outgroup. We concatenated 369 aligned single-copy coding sequences and obtained a matrix of 219,777 precise positions to construct a species phylogenetic tree (Fig. 3). The maximum likelihood and Bayesian inference methods generated highly consistent results, indicating the tree is robust. The newly constructed species tree will contribute to correct and improve the current taxonomy of *Paenibacillus*. The phylogenetic tree shows that *P. panacisoli* DSM 21345 and *P. massiliensis* DSM 16942 are likely the same species (16S similarity: 99.53%), *P*. sp. J14 should belong to *P. barengoltzii* (16S similarity: 99.32%), and *P. peoriae* HS311 (16S similarity: 99.49%) and *P*. sp. UNCCL52 (16S similarity: 99.69%) should belong to *P. polymyxa* (Fig. 3 and Dataset S2).

### Evolution of glycometabolism is crucial for *Paenibacillus* survival

Using a parsimony approach, the ancestral gene sets were reconstructed, and events leading to changes in the genomic content were identified and mapped to species tree (Fig. 3). We divided the change events into four categories: 1) gain, which denotes that a gene family was not present in the ancestral gene set and came into beings; 2) loss, which denotes that a gene family was present in the ancestral gene set and missed; 3) expansion, which denotes that the member of a gene family increased; 4) reduction, which denotes that the member of a gene family reduced (Figure S4). The results show the common ancestor of the genus *Paenibacillus* contained 1,833 gene families, which is larger than the size of core genome. It suggests that massive gain events occurring in every strain result in an increase of their genomic sizes. The occurrence frequency of loss is second, and lineage-specific loss events resulted in a decrease in the size of the core genome. The results indicate that gain and loss largely contributed to the difference in genomic content and genome size in *Paenibacillus*.

**Figure 4:**
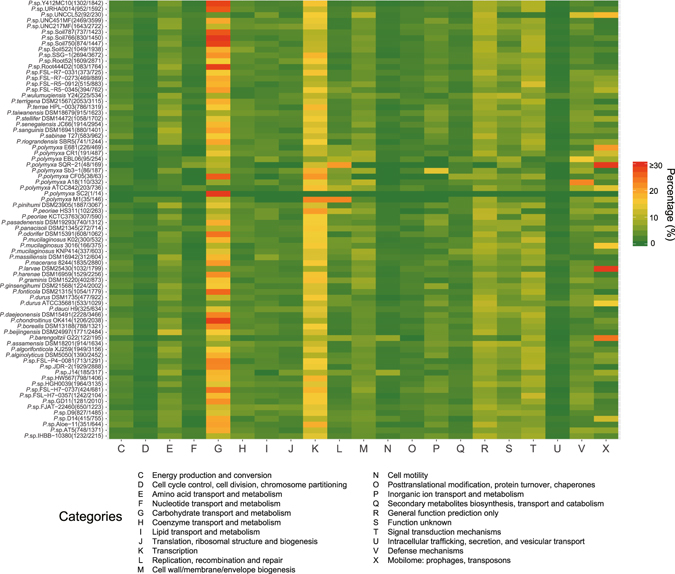
Functional distribution of the gained genes in each strain. The numbers listed after the strain name denote the number of COG assigned genes and total genes.

Gained genes are often more important for survival. To understand the roles of gained genes, we further investigated the functional distributions of the recently gained genes in each strain based on COG assignment (Fig. 4). The result shows that the gained genes are most abundant in carbohydrate transport and metabolism (Category G), following by transcription (Category K), corresponding to the functional distribution of pan-genome described above. This observation is different from the previous result that genes involved in regulation and secondary metabolism (Category K, T and Q) disproportionately expanded in larger genomes but genes associated with carbohydrate transport and metabolism (Category G) showed no significantly increase^30^. Expansion in Category K is not surprising, for increased genome and complex environmental conditions require more sophisticated transcriptional regulation systems^30^. The proteins encoded by gained genes of Category G can be divided into two parts: carbohydrate transporters and carbohydrate-active enzymes (CAZymes). ATP-binding cassette transporters (ABC transporters) and major facilitator superfamily transporters (MFS transporters) are two types of proteins that are responsible for transporting nutrients, including ions, amino acids and sugars, with representatives in both eukaryotes and prokaryotes^31, 32^. The phosphotransferase system transporters (PTS transporters) are involved in transporting many sugars into bacteria, and vary with different environments where the different carbon sources are available^33^. All three types of transporter genes are abundant in gained gene repertoires of *Paenibacillus* (Dataset S3). The CAZy database is currently the most authoritative CAZyme classification database^34^. The gained CAZymes of *Paenibacillus* were classified into 74 glycoside hydrolase families (GHs), 14 glycosyltransferase families (GTs), 7 polysaccharide lyase families (PLs) and 7 carbohydrate esterases families (CEs), covering a broad-spectrum of substrate activity (Dataset S3). The results indicate that *Paenibacillus* in different habitats evolved different abilities to utilize various carbon sources. For example, *Paenibacillus* sp. JDR-2 was isolated from the cut stems of sweet gum^35^, and it gained many xylose transport and metabolism related genes (Dataset S3). Seven strain were isolated from milk, and they gained many genes associated with lactose degradation (Dataset S1 and Dataset S3). Taken together, these observations indicate that the pattern of genome evolution is not fixed, and that the evolution of glycometabolism may be crucial to the survival of *Paenibacillus* species.

### Agar-degrading traits of *Paenibacillus* sp. SSG-1 conferred through horizontal gene transfer

**Figure 5:**
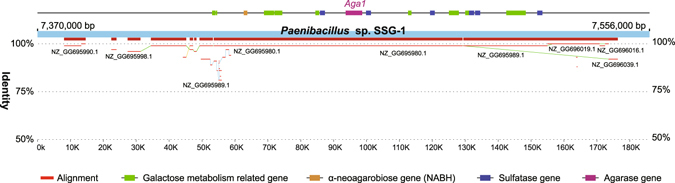
Match of whole-genome alignment between *Paenibacillus* sp. SSG-1 and D14 in the HGT region.

*Paenibacillus* sp. SSG-1 has the ability to degrade agar, a mixture of heterogeneous galactans. However, the agar-degrading traits are usually presented by marine organisms^36–38^. We proposed that the agarase gene *Aga1* together with surrounding genes in SSG-1 were acquired through HGT, but the evidences obtained in the previous study were limited^26^. In the pan-genome analysis, we found that *Aga1* were clustered with two hypothetic agarase genes in *Paenibacillus* sp. D14 and one of two is virtually identical with *Aga1*. Therefore, we implemented whole-genome alignment analyses between SSG-1 and D14. Interestingly, an almost identical region was detected in the two genomes and approximately locates at 7.40–7.55 M of the *Paenibacillus* sp. SSG-1 genome, which contains the comprehensively studied gene of *Aga1* (Fig. 5). Moreover, we detected abundant insert sequences and transposon sequences in this matching region (Fig. 1). These results indicate that D14 may also possess agar-degrading capacity and both strains may have acquired this trait through horizontal gene transfer.

**Figure 6:**
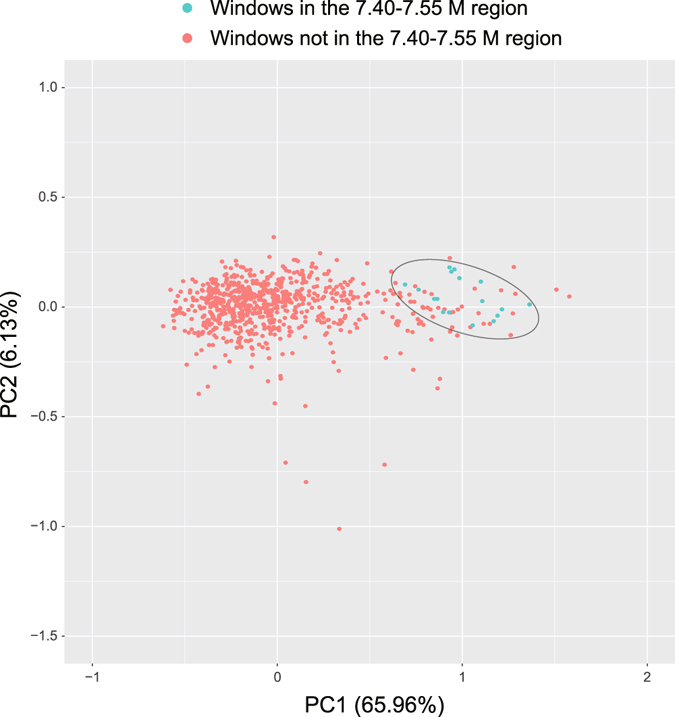
PCA analysis of codon usage frequency of *Paenibacillus* sp. SSG-1. The X and Y axes represent the scores of the first two principal components and each dot denotes 20 kb genome regions.

Subsequently, additional evidence of HGT was obtained. The 7.40–7.55 M region of the *Paenibacillus* sp. SSG-1 genome shows an obvious difference in GC content (mean GC: 0.4597) compared with the global genome (mean GC: 0.5305). We further calculated the frequency of codon usage in each region of the SSG-1 genome using a sliding window (20 kb window size; 10 kb step size). Then, a 61-dimensional matrix of codon usage frequency was obtained, and we performed the principal component analysis (PCA) for this matrix. The contribution of the principal component one (PC1) and the principal component two (PC2) reach 65.96% and 6.13%, respectively. Therefore, we used the scores of PC1 and PC2 for cluster analysis and result display. The results show that the windows located in 7.40–7.55 M region gathered but deviated from the main cluster (Fig. 6). It indicates that the codon usage bias of the genes located in 7.40–7.55 M region is different from that of most other genes (Fig. 6 and **fF S3**). Taken together, above results intensively support the inference that the 7.40–7.55 M region was inserted into the genome of *Paenibacillus* sp. SSG-1 through HGT.

Furthermore, we performed a GO enrichment analysis of genes located in the 7.40–7.55 M regions of the *Paenibacillus* sp. SSG-1 genome, and the significant functions include galactose metabolism (GO:0006012) and sulfuric ester hydrolase activity (GO:0008484) (Table S4). Agar consists of alternating 3-O-linked β-D galactopyranose (G) and 4-O-linked α-L-galactopyranose (LA) linked to sulphate or other groups^39^. Agar degradation involved multiple process: sulfatases hydrolyze the sulphate ester in agar to transform sulphated agar into agarose; agarases degrade agarose into agaro-oligosaccharides; agaro-oligosaccharides are further hydrolysed by α-neoagarobiose hydrolases (NABH) into the monomers G and LA, and finally utilized by galactose metabolic processes^39, 40^. Complete pathway and abundant genes related to agar degradation were found in 7.40–7.55 M region (Fig. 5), suggesting that this region as a complete unit responsible for agar degradation merged into the *Paenibacillus* sp. SSG-1 genome.

## Discussion

The genome size of microorganisms influences their extensive adaptabilities to the environment. Under most conditions, strains with larger genome size generally may be more adaptive to complex habitats, as these microorganisms may encode more products for metabolism and stress tolerance^41^. However, some studies have suggested that the small size of bacterial genomes may be also competitive, reflecting advantages in energy saving and reproductive efficiency^42, 43^. In summary, there is a balance between maintaining a minimum genome size and facilitating the response to environmental conditions.

The genus *Paenibacillus* exhibits extensive environmental adaptability and can populate various ecological niches. To our knowledge, this study is the first to investigate the pan-genome of the genus *Paenibacillus* and explore the evolutionary reason for the wide niche adaptation of these bacteria. The results show that the pan-genome of the genus *Paenibacillus* is open and theoretically infinite, suggesting that *Paenibacillus* species tend to acquire new genes to enhance the adaptability. In contrast to increasing pan-genome, the core genome was decreasing, suggesting that the difference in genomic content in *Paenibacillus* was gradually increasing. Free-living bacteria typically have an open pan-genome whose size shows a tendency toward continuous growth^44^. Bacteria must change their genetic material to adapt to variable environmental conditions, thus, greater niche diversity reflects larger pan-genomes^30, 45^. Considering the wide distribution of *Paenibacillus*, a large pan-genome size corresponds to diverse living conditions.

The gene repertoires of bacteria are dynamic, and the incessant flux reflects four major events: gain, loss, expansion and reduction (Figure S4). The events of gain and loss occurred frequently in the evolution of *Paenibacillus* species. The present results show that the gene repertoires of the genus *Paenibacillus* are in incessant flux and the genome size of *Paenibacillus* shows an increasing tendency. As described above, to reduce energy consumption, *Paenibacillus* species abandoned some dispensable genes while acquiring new characters. The events of gain and loss during evolution largely contributed to the difference in genomic content and genome size. Gained genes are more important for survival. *Paenibacillus* species gained a large number of genes associated with carbohydrate transport and metabolism, which differ from the most other bacteria^30^. After a long time of evolution, although the core genome became very small, it still contains a large number of genes associated with flagellar movement. These results indicate that active movement and sugar uptake are the keys to the survival of *Paenibacillus*. For different habitats, carbon sources which microorganisms can directly use are so diverse that bacteria must evolve their glycometabolism ability to ensure environmental adaptability. Hence, the broad environmental adaptability of *Paenibacillus* may be caused by abundant glycometabolism-related genes.

The main approaches of gain include horizontal gene transfer (HGT) and gene diversification, and the former is more widespread in bacteria^46^. The agar-degrading traits of *Paenibacillus* sp. SSG-1 may largely reflect HGT. The methods used to detect HGT are primarily divided into two categories: parametric and phylogenetic methods^47^. The parametric method is based on genomic features, and foreign gene regions are typically show differences in GC-content, codon usage, tetra-nucleotide frequency and other attributes. However, these methods are limited by recent HGT events, as the alien features are assimilated with time^48^. The phylogenetic methods explore the evolutionary histories of genes involved and identify conflicting phylogenies, i.e., considering a well-established species tree and a gene tree, and the inconsistencies in some nodes may indicate occurrences of HGT. In the present study, a large HGT region in *Paenibacillus* sp. SSG-1 genome was confirmed using various methods. The HGT region endows SSG-1 an agar-degrading capacity which is distinct from most other non-marine bacteria. In addition, a homologous HGT region was observed in *Paenibacillus* sp. D14. According to the phylogenetic tree, SSG-1 is far from D14 and their closely related strains do not possess this agar-degrading region (Fig. 3). Therefore, we suggested the following hypothetical HGT pathways for these two regions in SSG-1 and D14: first, one common ancestor provided this region separately inserted into SSG-1 and D14’ genomes; second, one of SSG-1 or D14 acquired this region through HGT, and subsequently transferred this genetic material to another strain. In either case, the two agar-degrading regions of SSG-1 and D14 show the same origin during evolution. However, the issue who is the donor or where is the origin of this region still is a puzzle. We attempted to blast this region against the nr/nt, refseq genomic and gss databases using the NCBI online service, but no other remarkable match was observed. Considering the origin of soil-isolated agarase, as previously discussed^26^, this region is most likely derived from marine organisms.

## Conclusions

To our knowledge, this study is the first to investigate the pan-genome of the genus *Paenibacillus* and explore the evolutionary reason for the wide niche adaptation of these microorganisms. The pan-genome of *Paenibacillus* is open and members in this genus tend to change their genomic contents to adapt to the environment. The events of gain and loss during evolution largely contributed to the difference in the genomic content and the genome size. The evolution of glycometabolism is a key factor for the environmental adaptability of the genus *Paenibacillus*. Moreover, the core-genome phylogeny contributed to taxonomy in the genus *Paenibacillus*.

## Materials and Methods

### Genome sequencing and data acquisition

For *Paenibacillus* sp. SSG-1, strain culture and genome DNA extraction were the same as previously reported^25^. Genome DNA was fragmented, and two DNA libraries were constructed (~500 and ~6,000 bp). Whole-genome sequencing was performed using a high-throughput Illumina HiSeq 2000 sequencing platform at BGI-Shenzhen, Shenzhen, China. After cleaning, the paired-end reads were assembled using SOAPdenovo v2.04^49^, and 754 Mb of the read sequences represented a 100-fold coverage of the genome. Structures of protein-coding genes, structural RNAs (5S, 16S, 23S), tRNAs and small non-coding RNAs were predicted using NCBI Prokaryotic Genome Annotation Pipeline (PGAP)^50^. The cleaned sequencing reads were deposited in the SRA database under accession number SRR3948181, and the genome sequence was deposited in GenBank under accession number MBRK00000000.

Other strain genomes and annotation information used in the present study were downloaded from the NCBI refseq genomic database and were summarized in Dataset S1.

### Filtering and assessment of gene sets

In order to ensure the reliability of subsequent analysis, the gene sets of all strains were strictly filtered. We considered the screening criteria: 1) protein-coding genes encoding less than 50 aa were filtered, 2) genes which contained an inside stop codon in ORF were filtered, and 3) genes with a high proportion of N (>30%) were also filtered.

The completeness and quality of filtered gene sets was assessed using BUSCO v2.0^27^ based on evolutionarily informed expectations of gene content. We used two BUSCO lineage datasets for our assessments: firmicutes_odb9 containing 232 BUSCOs and bacillales_odb9 containing 526 BUSCOs.

### Functional annotation and analysis

Annotation of protein coding sequence was performed by blasting to functional databases, including NCBI non-redundant (NR, filtered unknown and hypothetical proteins), SwissProt^51^, and enhanced COG database^52^. Poor alignments were removed, and the highest quality alignments were selected as gene annotations. Gene ontology (GO) terms were assigned using Blast2GO software^53^. KEGG annotation was implemented using online BlastKOALA^54^. Carbohydrate-active enzymes^34^ were classified into families using online dbCAN annotation server^55^.

GO enrichment analyses were performed using the R package topGO^56^, and the P-values were calculated using Fisher’s exact test and corrected using Benjamini-Hochberg method. The GO term is significant when corrected P-values ≤ 0.05.

### Pan-genome analysis

Proteins of all strains were reciprocally compared using blastp v2.2.31+, and poor alignments were removed. Subsequently, the proteins were classified into families using OrthoMCL v2.0.9^57^ with an inflation index of 1.5. The core, single-copy and unique families were identified according to the classification results. Gene accumulation curves, describing the number of core and pan gene families, with the addition of new comparative genomes were performed using a self-writing R script for parsing OrthoMCL results. This procedure was repeated 1,000 times for every addition of a randomly selected genome to obtain median values.

### Phylogenetic analysis

The proteins were aligned using muscle v3.8.425^58^ per single-copy family, followed by transformation to a CDS alignment using pal2nal v14^59^. Subsequently, we used Gblocks v0.91b^60^ to extract unambiguous parts and concatenated all family alignments. We used this alignment for further phylogenetic analysis. RAxML v8.2.7^61^ and MrBayes v3.2.4^62^ were used to construct phylogenetic trees. For RAxML, we selected a GTR substitutions model with an estimated proportion of gamma distribution and invariant sites, and set 1,000 bootstrap iterations for calculation. For MrBayes, we selected the same nucleotide substitution model and set 1,000,000 iterations for MCMC, and trees were sampled every 100 iterations with the first 2,500 samples as burn-in.

The 16S rDNA sequences were obtained according to annotation information. The pairwise identity of 16S rDNA was obtained using blastn and summaried in Dataset S2. The sequences with high integrity (>1,000 bp) were used for phylogenetic analysis. A clustal alignment algorithm and neighbor-joining clustering method were implemented in MEGA v7^63^.

### Genomic content change

The ancestral gene sets were reconstructed using Count v10.04^64^ based on the Wagner parsimony algorithm with a gain penalty of one. Events leading to changes in the genomic content (gain, loss, expansion and reduction) were identified after comparing each node with its latest ancestor.

### Inference of horizontal gene transfer

Whole genome alignments were performed using the nucmer script in the MUMmer v3.23 software package^65^ with *Paenibacillus* sp. SSG-1 as the reference genome. Alignment maps were drawn using the mapview script in the MUMmer package. Insert and transposon sequences were detected using ISFinder^66^. The GC content per 5 kb was calculated using self-writing Perl script. The circular map of *Paenibacillus* sp. SSG-1 was drawn using Circos v0.67.7^67^.

The codon usage analysis was performed according to the following steps: A 20 kb sliding window with 10 kb step size was scanning the genome of *Paenibacillus* sp. SSG-1, and the codon usage frequencies (FCU, calculated using self-writing Perl script) of the genes located in each window (overlapped >50%) were calculated. Therefore, a 61-dimension matrix was generated, and every dimension described one FCU per codon. We subsequently performed a principal component analysis (PCA) for this matrix using R, and the scores of the first two compositions were used to exhibit the results. In addition, a general comparison of codon usage between the 7.40–7.55 M region with the global genome was also shown in Table S3.

## Electronic supplementary material

Supplementary Information

Supplementary Dataset
