# Supplementary material for: Comparative genomic analysis of Paenibacillus sp. SSG-1 and its closely related strains reveals the effect of glycometabolism on environmental adaptation
Source: Sci Rep. 2017 Jul 18;7:5720. doi: 10.1038/s41598-017-06160-9 (PMC5516027; doi:10.1038/s41598-017-06160-9)
Supplement: Supplementary file 1 — Supplementary Information [file 41598_2017_6160_MOESM1_ESM.pdf]

Supplementary Information for

Comparative genomic analysis of *Paenibacillus* sp. SSG-1 and its closely related strains reveals the effect of glycometabolism on environmental adaptation

Hui Xu[1+], Shishang Qin[1+], Yanhong Lan[1], Mengjia Liu[1], Xiyue Cao[2], Dairong Qiao[1], Yu Cao[1]*, Yi Cao[1]*

[1]Microbiology and Metabolic Engineering of Key Laboratory of Sichuan Province, College of Life Science, Sichuan University, Chengdu, 610065, P. R. China

[2]College of Food Science, Northeast Agricultural University, Harbin, 150030, P. R. China

[+]These authors contributed equally to this work

[*]Corresponding author

Correspondence addresses: geneium@scu.edu.cn, caoyu1984@163.com

Contents

Table S1. The completeness assignment of gene sets used in the present study.

Table S2. The GO function enrichment of *Paenibacillus* sp. SSG-1 genes in core genome.

Table S3. General comparison of codon usage between the 7.40-7.55 M region with the global genome of *Paenibacillus* sp. SSG-1.

Table S4. The GO function enrichment of *Paenibacillus* sp. SSG-1 genes located in the 7.40-7.55 M region.

Figure S1. Clustering distribution of genes in each strain.

Figure S2. Overview of functional distribution based on COG assignment.

Figure S3. The 16S tree of *Paenibacillus* based on the neighbour-joining method.

Figure S4. Schematic representation of gain, lose, expansion and reduction.

In addition, we provided following datasets in another separate Excel file.

Dataset S1. The information on the strains used in the present study.

Dataset S2. The pairwise identity of 16S rDNA.

Dataset S3. Functional annotation of the gained glycometabolism-related genes in each strain.

Table S1. The completeness assignment of gene sets used in the present study.

Strain	Lineage dataset: firmicutes_odb9 (232 BUSCOs)					Lineage dataset: bacillales_odb9 (526 BUSCOs)				
	%C	%S	%D	%F	%M	%C	%S	%D	%F	%M
P. sp. SSG-1	98.80	96.60	2.20	0.40	0.80	97.50	95.20	2.30	1.00	1.50
P. sp. IHBB-10380	99.50	97.80	1.70	0.40	0.10	95.80	93.50	2.30	1.50	2.70
P. sp. AT5	97.90	96.60	1.30	0.40	1.70	95.00	93.30	1.70	1.70	3.30
P. sp. Aloe-11	96.10	95.70	0.40	1.70	2.20	92.80	90.90	1.90	1.70	5.50
P. sp. D14	97.00	96.10	0.90	0.90	2.10	96.00	94.90	1.10	1.10	2.90
P. sp. D9	97.00	95.70	1.30	0.40	2.60	94.20	93.20	1.00	2.50	3.30
P. sp. FJAT-22460	99.20	98.30	0.90	0.40	0.40	96.80	94.90	1.90	1.00	2.20
P. sp. GD11	97.90	96.60	1.30	0.00	2.10	93.30	91.60	1.70	1.30	5.40
P. sp. FSL-H7-0357	99.60	98.70	0.90	0.40	0.00	96.90	95.80	1.10	1.50	1.60
P. sp. FSL-H7-0737	100.00	99.10	0.90	0.00	0.00	96.50	95.20	1.30	1.70	1.80
P. sp. HGH0039	97.80	97.40	0.40	0.40	1.80	97.50	95.20	2.30	1.00	1.50
P. sp. HW567	100.00	99.60	0.40	0.00	0.00	97.10	95.60	1.50	1.10	1.80
P. sp. J14	99.50	99.10	0.40	0.00	0.50	98.10	97.70	0.40	0.80	1.10
P. sp. JDR-2	99.50	99.10	0.40	0.00	0.50	95.30	93.00	2.30	1.70	3.00
P. sp. FSL-P4-0081	99.20	98.30	0.90	0.40	0.40	96.20	94.70	1.50	1.90	1.90
P. *alginolyticus* DSM5050	98.70	97.80	0.90	0.40	0.90	97.90	95.40	2.50	0.80	1.30
P. *algorifonticola* XJ259	98.70	97.80	0.90	0.40	0.90	96.40	95.10	1.30	2.10	1.50
P. *assamensis* DSM18201	99.50	99.10	0.40	0.00	0.50	96.20	95.40	0.80	1.30	2.50
P. *barengoltzii* G22	99.50	99.10	0.40	0.00	0.50	97.70	97.30	0.40	1.00	1.30
P. *beijingensis* DSM24997	98.30	96.60	1.70	0.90	0.80	95.40	92.40	3.00	1.50	3.10
P. *borealis* DSM13188	99.20	98.30	0.90	0.90	-0.10	95.80	93.90	1.90	2.30	1.90
P. *chondroitinus* OK414	99.20	98.30	0.90	0.40	0.40	98.10	94.30	3.80	1.00	0.90
P. *daejeonensis* DSM15491	100.00	99.10	0.90	0.00	0.00	96.20	94.50	1.70	1.30	2.50
P. *dauci* H9	99.60	98.30	1.30	0.00	0.40	95.80	94.70	1.10	2.10	2.10

P. durus ATCC35681	99.10	98.70	0.40	0.90	0.00	96.30	95.20	1.10	2.50	1.20
P. durus DSM1735	98.70	97.80	0.90	0.90	0.40	96.70	95.20	1.50	1.90	1.40
P. fonticola DSM21315	99.50	99.10	0.40	0.00	0.50	96.60	95.10	1.50	1.10	2.30
P. ginsengihumi DSM21568	97.90	96.60	1.30	1.70	0.40	94.50	92.80	1.70	2.30	3.20
P. graminis DSM15220	100.00	99.10	0.90	0.00	0.00	96.40	95.10	1.30	2.30	1.30
P. harenae DSM16959	99.50	97.80	1.70	0.00	0.50	96.40	94.50	1.90	1.10	2.50
P. larvae DSM25430	97.00	96.10	0.90	1.30	1.70	95.50	94.90	0.60	1.70	2.80
P. macerans 8244	98.30	97.40	0.90	0.00	1.70	96.20	93.90	2.30	1.00	2.80
P. massiliensis DSM16942	100.00	99.60	0.40	0.00	0.00	97.10	95.80	1.30	1.70	1.20
P. mucilaginosus KNP414	97.40	96.10	1.30	0.40	2.20	96.60	94.70	1.90	1.70	1.70
P. mucilaginosus 3016	93.60	92.70	0.90	1.30	5.10	92.80	91.10	1.70	1.90	5.30
P. mucilaginosus K02	98.30	97.00	1.30	0.40	1.30	97.00	94.90	2.10	1.70	1.30
P. odorifer DSM15391	100.00	99.60	0.40	0.00	0.00	96.80	96.00	0.80	1.30	1.90
P. panacisoli DSM21345	99.50	99.10	0.40	0.00	0.50	97.30	96.20	1.10	1.50	1.20
P. pasadenensis DSM19293	97.80	97.40	0.40	0.90	1.30	95.10	93.00	2.10	2.50	2.40
P. peoriae KCTC3763	99.60	98.70	0.90	0.40	0.00	97.50	96.40	1.10	1.10	1.40
P. peoriae HS311	100.00	98.70	1.30	0.00	0.00	97.40	94.90	2.50	1.50	1.10
P. pinihumi DSM23905	100.00	98.70	1.30	0.00	0.00	95.60	93.30	2.30	1.70	2.70
P. polymyxa M1	99.60	98.70	0.90	0.40	0.00	97.50	95.80	1.70	1.50	1.00
P. polymyxa SC2	99.60	98.70	0.90	0.40	0.00	97.30	95.60	1.70	1.50	1.20
P. polymyxa ATCC842	99.60	98.30	1.30	0.40	0.00	97.70	95.80	1.90	1.30	1.00
P. polymyxa A18	99.60	98.70	0.90	0.40	0.00	97.50	96.20	1.30	1.30	1.20
P. polymyxa CF05	99.60	98.30	1.30	0.40	0.00	97.50	95.60	1.90	1.50	1.00
P. polymyxa Sb3-1	99.50	99.10	0.40	0.40	0.10	97.60	96.60	1.00	1.30	1.10
P. polymyxa SQR-21	99.10	97.80	1.30	0.40	0.50	97.10	95.20	1.90	1.50	1.40
P. polymyxa EBL06	99.60	98.30	1.30	0.40	0.00	97.30	95.20	2.10	1.50	1.20
P. polymyxa CR1	98.30	97.00	1.30	0.40	1.30	94.10	91.60	2.50	1.70	4.20
P. polymyxa E681	99.60	98.70	0.90	0.00	0.40	97.30	95.60	1.70	1.50	1.20

P. riograndensis SBR5	100.00	99.10	0.90	0.00	0.00	96.80	95.10	1.70	1.90	1.30
P. sabinae T27	97.90	96.60	1.30	0.40	1.70	96.20	94.70	1.50	1.90	1.90
P. sanguinis DSM16941	100.00	99.60	0.40	0.00	0.00	95.60	94.30	1.30	1.70	2.70
P. senegalensis JC66	97.90	97.00	0.90	0.40	1.70	94.90	93.20	1.70	1.50	3.60
P. stellifer DSM14472	99.20	98.30	0.90	0.90	-0.10	96.90	95.80	1.10	1.90	1.20
P. taiwanensis DSM18679	100.00	99.60	0.40	0.00	0.00	97.00	96.00	1.00	1.70	1.30
P. terrae HPL-003	98.70	98.30	0.40	0.40	0.90	96.00	93.90	2.10	1.70	2.30
P. terrigena DSM21567	99.60	98.70	0.90	0.00	0.40	96.80	95.10	1.70	1.70	1.50
P. wulumuqiensis Y24	99.60	98.30	1.30	0.00	0.40	96.40	94.90	1.50	1.90	1.70
P. sp. FSL-R5-0345	100.00	99.10	0.90	0.00	0.00	96.70	95.60	1.10	1.50	1.80
P. sp. FSL-R5-0912	100.00	99.10	0.90	0.00	0.00	96.40	94.70	1.70	1.90	1.70
P. sp. FSL-R7-0273	99.50	99.10	0.40	0.40	0.10	96.60	96.00	0.60	1.50	1.90
P. sp. FSL-R7-0331	99.50	99.10	0.40	0.40	0.10	96.00	95.20	0.80	1.90	2.10
P. sp. Root444D2	98.70	97.80	0.90	0.40	0.90	97.10	93.90	3.20	1.30	1.60
P. sp. Root52	99.50	97.80	1.70	0.00	0.50	96.60	95.80	0.80	1.90	1.50
P. sp. Soil522	99.20	98.30	0.90	0.40	0.40	96.80	94.90	1.90	1.30	1.90
P. sp. Soil750	98.30	97.00	1.30	0.40	1.30	96.80	94.50	2.30	1.30	1.90
P. sp. Soil766	99.10	97.80	1.30	0.40	0.50	97.50	95.20	2.30	1.00	1.50
P. sp. Soil787	98.70	97.80	0.90	0.40	0.90	98.10	95.20	2.90	0.80	1.10
P. sp. UNC217MF	100.00	98.70	1.30	0.00	0.00	98.10	96.20	1.90	1.50	0.40
P. sp. UNC451MF	99.10	97.80	1.30	0.40	0.50	97.40	94.70	2.70	0.80	1.80
P. sp. UNCCL52	100.00	98.70	1.30	0.00	0.00	97.70	95.20	2.50	1.30	1.00
P. sp. URHA0014	98.70	97.40	1.30	0.40	0.90	97.00	94.50	2.50	1.10	1.90
P. sp. Y412MC10	99.10	97.80	1.30	0.00	0.90	97.70	96.40	1.30	0.80	1.50
B. subtilis 168	99.50	99.10	0.40	0.40	0.10	99.40	99.40	0.00	0.00	0.60

%C: the percentage of complete BUSCOs; %S: the percentage of complete and singlecopy BUSCOs; %D: the percentage of complete and duplicated BUSCOs; %F: the percentage of fragmented BUSCOs; %M: the percentage of missing BUSCOs.

Table S2. The GO function enrichment of *Paenibacillus* sp. SSG-1 genes in core genome.

GO	Class	Corrected P-value	Term
GO:0003735	MF	0.0000	structural constituent of ribosome
GO:0019843	MF	0.0000	rRNA binding
GO:0003723	MF	0.0000	RNA binding
GO:0000049	MF	0.0000	tRNA binding
GO:0000287	MF	0.0000	magnesium ion binding
GO:0005524	MF	0.0000	ATP binding
GO:0016884	MF	0.0001	carbon-nitrogen ligase activity, with glutamine as amido-N-donor
GO:0016597	MF	0.0001	amino acid binding
GO:0005525	MF	0.0001	GTP binding
GO:0046872	MF	0.0044	metal ion binding
GO:0003899	MF	0.0046	DNA-directed RNA polymerase activity
GO:0046933	MF	0.0060	proton-transporting ATP synthase activity, rotational mechanism
GO:0009982	MF	0.0060	pseudouridine synthase activity
GO:0016765	MF	0.0141	transferase activity, transferring alkyl or aryl (other than methyl) groups
GO:0003924	MF	0.0179	GTPase activity
GO:0008175	MF	0.0253	tRNA methyltransferase activity
GO:0016881	MF	0.0266	acid-amino acid ligase activity
GO:0008649	MF	0.0371	rRNA methyltransferase activity
GO:0016624	MF	0.0371	oxidoreductase activity, acting on the aldehyde or oxo group of donors, disulfide as acceptor
GO:0003678	MF	0.0371	DNA helicase activity
GO:0004812	MF	0.0416	aminoacyl-tRNA ligase activity
GO:0050661	MF	0.0433	NADP binding
GO:0008135	MF	0.0480	translation factor activity, RNA binding
GO:0042254	BP	0.0000	ribosome biogenesis
GO:0006412	BP	0.0000	translation
GO:0006144	BP	0.0001	purine nucleobase metabolic process
GO:0006364	BP	0.0001	rRNA processing

GO:0044205	BP	0.0001	'de novo' UMP biosynthetic process
GO:0008654	BP	0.0001	phospholipid biosynthetic process
GO:0006206	BP	0.0017	pyrimidine nucleobase metabolic process
GO:0006189	BP	0.0018	'de novo' IMP biosynthetic process
GO:0009094	BP	0.0026	L-phenylalanine biosynthetic process
GO:0042777	BP	0.0032	plasma membrane ATP synthesis coupled proton transport
GO:0006094	BP	0.0032	gluconeogenesis
GO:0016226	BP	0.0032	iron-sulfur cluster assembly
GO:0006096	BP	0.0042	glycolytic process
GO:0046656	BP	0.0042	folic acid biosynthetic process
GO:0000162	BP	0.0050	tryptophan biosynthetic process
GO:0006571	BP	0.0050	tyrosine biosynthetic process
GO:0006281	BP	0.0057	DNA repair
GO:0006536	BP	0.0057	glutamate metabolic process
GO:0001522	BP	0.0057	pseudouridine synthesis
GO:0006730	BP	0.0057	one-carbon metabolic process
GO:0006260	BP	0.0057	DNA replication
GO:0009306	BP	0.0065	protein secretion
GO:0015940	BP	0.0090	pantothenate biosynthetic process
GO:0008033	BP	0.0115	tRNA processing
GO:0006457	BP	0.0115	protein folding
GO:0019643	BP	0.0115	reductive tricarboxylic acid cycle
GO:0090502	BP	0.0115	RNA phosphodiester bond hydrolysis, endonucleolytic
GO:0051252	BP	0.0147	regulation of RNA metabolic process
GO:0015994	BP	0.0159	chlorophyll metabolic process
GO:0006400	BP	0.0189	tRNA modification
GO:0071973	BP	0.0189	bacterial-type flagellum-dependent cell motility
GO:0006526	BP	0.0207	arginine biosynthetic process
GO:0046653	BP	0.0207	tetrahydrofolate metabolic process
GO:0006522	BP	0.0250	alanine metabolic process

GO:0006418	BP	0.0288	tRNA aminoacylation for protein translation
GO:0043244	BP	0.0343	regulation of protein complex disassembly
GO:0019856	BP	0.0343	pyrimidine nucleobase biosynthetic process
GO:0006935	BP	0.0381	chemotaxis
GO:0044780	BP	0.0381	bacterial-type flagellum assembly
GO:0006119	BP	0.0381	oxidative phosphorylation
GO:0009097	BP	0.0438	isoleucine biosynthetic process
GO:0032259	BP	0.0438	methylation
GO:0000902	BP	0.0468	cell morphogenesis
GO:0005737	CC	0.0000	cytoplasm
GO:0005840	CC	0.0000	ribosome
GO:0015935	CC	0.0001	small ribosomal subunit
GO:0043234	CC	0.0014	protein complex
GO:0005730	CC	0.0086	nucleolus
GO:0015934	CC	0.0127	large ribosomal subunit
GO:0044464	CC	0.0262	cell part
GO:1990234	CC	0.0370	transferase complex
GO:0044424	CC	0.0370	intracellular part
GO:0045261	CC	0.0370	proton-transporting ATP synthase complex, catalytic core F(1)
GO:0070013	CC	0.0424	intracellular organelle lumen

Table S3. General comparison of codon usage between the 7.40-7.55 M region with the global genome of *Paenibacillus* sp. SSG-1.

Amino acid	Code	Frequency		RSCU	
		Global	7.40-7.55 M	Global	7.40-7.55 M
A	GCA	0.1530	0.2890	0.6121	1.1561
	GCC	0.3686	0.1984	1.4745	0.7936
	GCG	0.3312	0.2519	1.3247	1.0077
	GCT	0.1472	0.2607	0.5887	1.0427
C	TGC	0.7639	0.4888	1.5277	0.9777
	TGT	0.2361	0.5112	0.4723	1.0223
D	GAC	0.5081	0.2901	1.0163	0.5803
	GAT	0.4919	0.7099	0.9837	1.4197
E	GAA	0.5048	0.5272	1.0096	1.0544
	GAG	0.4952	0.4728	0.9904	0.9456
F	TTC	0.5065	0.4914	1.0131	0.9827
	TTT	0.4935	0.5086	0.9869	1.0173
G	GGA	0.2221	0.2819	0.8884	1.1274
	GGC	0.4325	0.3014	1.7300	1.2057
	GGG	0.2043	0.1594	0.8171	0.6377
	GGT	0.1411	0.2573	0.5645	1.0292
H	CAC	0.3693	0.2533	0.7387	0.5066
	CAT	0.6307	0.7467	1.2613	1.4934
I	ATA	0.0690	0.1498	0.2071	0.4495
	ATC	0.5685	0.3249	1.7056	0.9748
	ATT	0.3624	0.5252	1.0873	1.5757
K	AAA	0.4621	0.4461	0.9243	0.8921
	AAG	0.5379	0.5539	1.0757	1.1079
L	CTA	0.0275	0.0798	0.1651	0.4787
	CTC	0.1555	0.0876	0.9327	0.5256
	CTG	0.4910	0.2609	2.9460	1.5655

	CTT	0.1247	0.1978	0.7482	1.1871
	TTA	0.0570	0.1237	0.3422	0.7423
	TTG	0.1443	0.2501	0.8659	1.5008
M	ATG	1.0000	1.0000	1.0000	1.0000
N	AAC	0.6022	0.3626	1.2045	0.7251
	AAT	0.3978	0.6374	0.7955	1.2749
P	CCA	0.0959	0.2415	0.3837	0.9661
	CCC	0.1697	0.1144	0.6788	0.4577
	CCG	0.5726	0.3692	2.2903	1.4767
	CCT	0.1618	0.2749	0.6472	1.0995
Q	CAA	0.2944	0.4910	0.5889	0.9820
	CAG	0.7056	0.5090	1.4111	1.0180
R	AGA	0.0893	0.1867	0.5358	1.1204
	AGG	0.0839	0.0864	0.5035	0.5185
	CGA	0.0626	0.1677	0.3757	1.0062
	CGC	0.2947	0.1862	1.7681	1.1173
	CGG	0.3353	0.1651	2.0120	0.9907
	CGT	0.1342	0.2078	0.8050	1.2469
S	AGC	0.2980	0.2075	1.7882	1.2448
	AGT	0.0659	0.1633	0.3951	0.9799
	TCA	0.0674	0.1264	0.4041	0.7584
	TCC	0.2636	0.1790	1.5815	1.0738
	TCG	0.2295	0.1653	1.3769	0.9920
	TCT	0.0757	0.1585	0.4541	0.9510
T	ACA	0.1283	0.3041	0.5130	1.2163
	ACC	0.3348	0.1888	1.3394	0.7554
	ACG	0.4575	0.3306	1.8300	1.3224
	ACT	0.0794	0.1765	0.3176	0.7060
V	GTA	0.1453	0.2426	0.5814	0.9705
	GTC	0.3286	0.1857	1.3144	0.7430

	GTG	0.3520	0.2973	1.4082	1.1891
	GTT	0.1740	0.2744	0.6961	1.0975
W	TGG	1.0000	1.0000	1.0000	1.0000
Y	TAC	0.4927	0.3256	0.9853	0.6511
	TAT	0.5073	0.6744	1.0147	1.3489

RSCU: relative synonymous codon usage; Global CDS GC3s:0.6630; 7.40-7.55 M region CDS GC3s:0.4844.

Table S4. The GO function enrichment of *Paenibacillus* sp. SSG-1 genes located in the 7.40-7.55 M region.

**GO**	**Class**	**Corrected P-value**	**Term**
GO:0004565	MF	0.0085	beta-galactosidase activity
GO:0008484	MF	0.0391	sulfuric ester hydrolase activity
GO:0006012	BP	0.0111	galactose metabolic process
GO:0006687	BP	0.0177	glycosphingolipid metabolic process
GO:0006027	BP	0.0177	glycosaminoglycan catabolic process
GO:0009341	CC	0.0009	beta-galactosidase complex

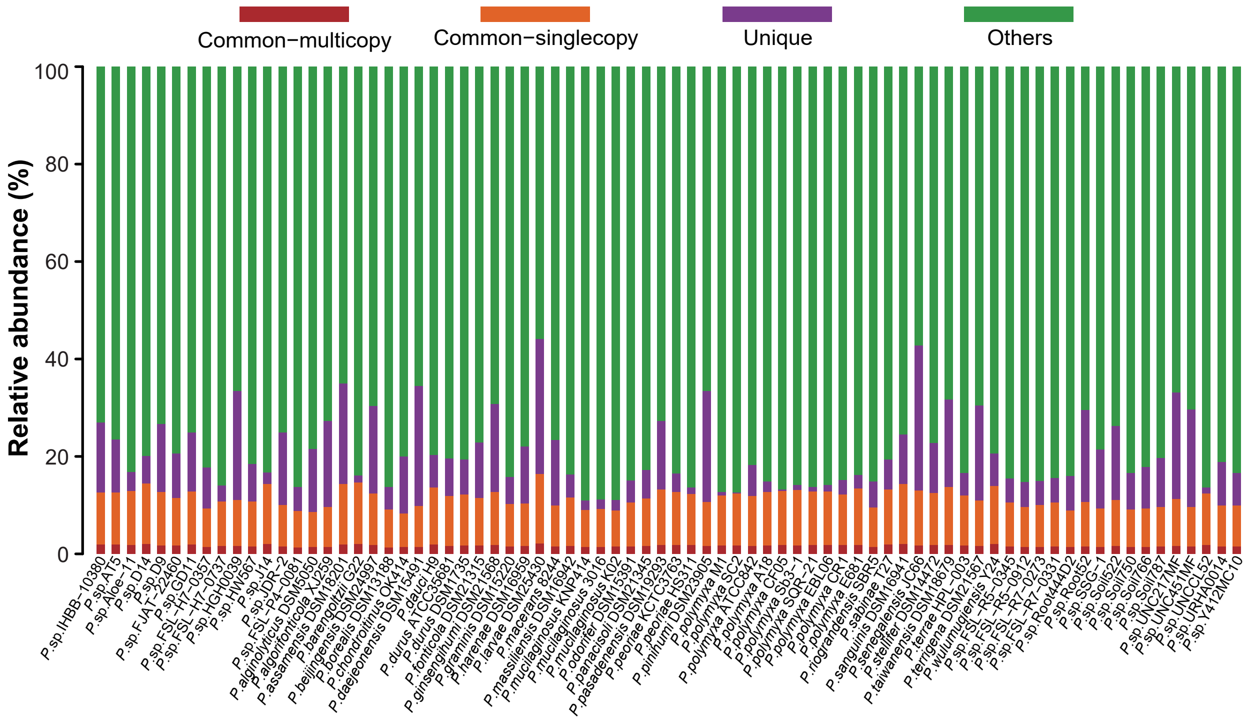

Figure S1. Clustering distribution of genes in each strain.

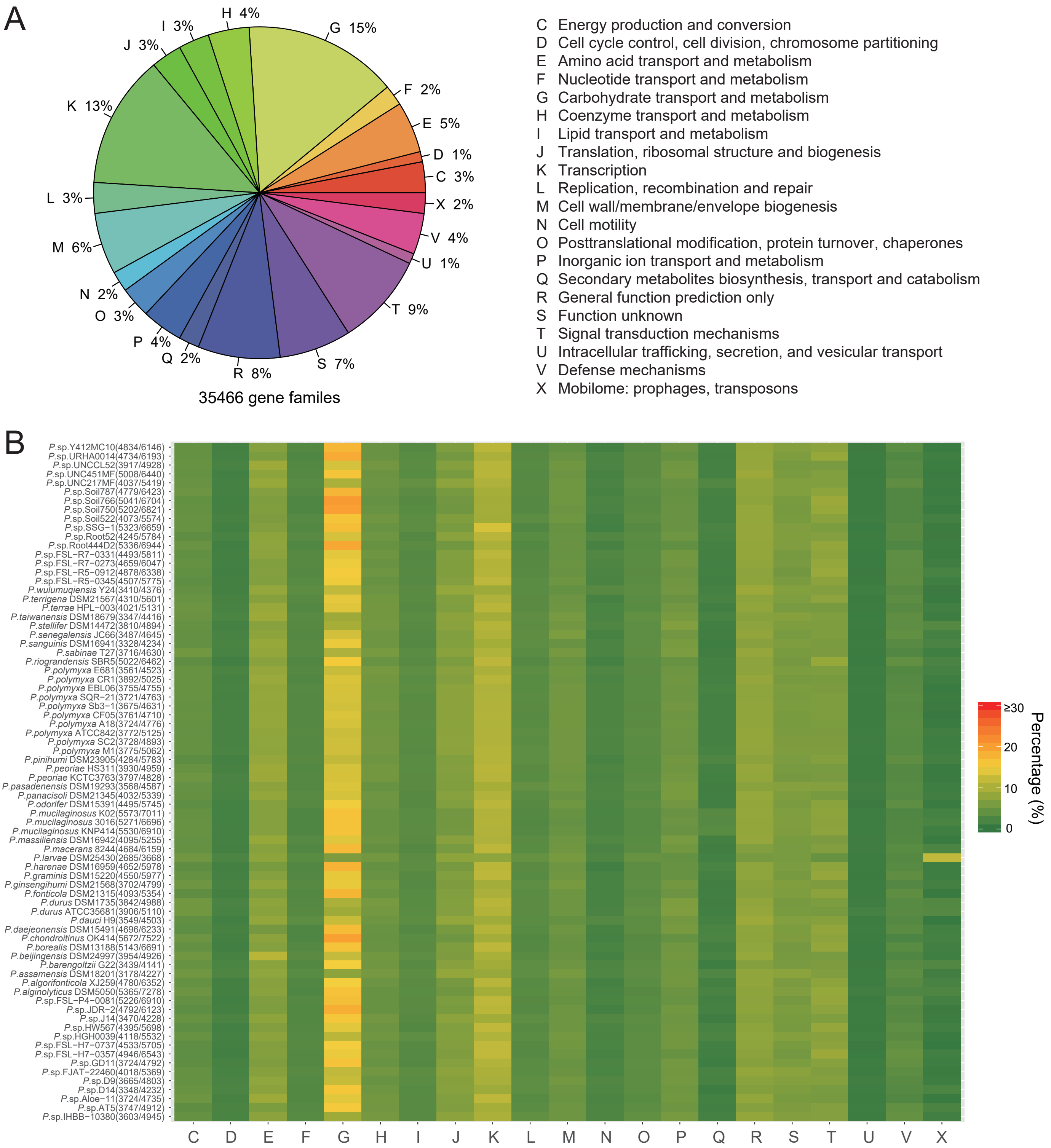

Figure S2. Overview of functional distribution based on COG assignment.
(A) Functional distribution of the COG assigned gene families in the pan-genome.
(B) Functional distribution of the COG assigned genes in each strain.

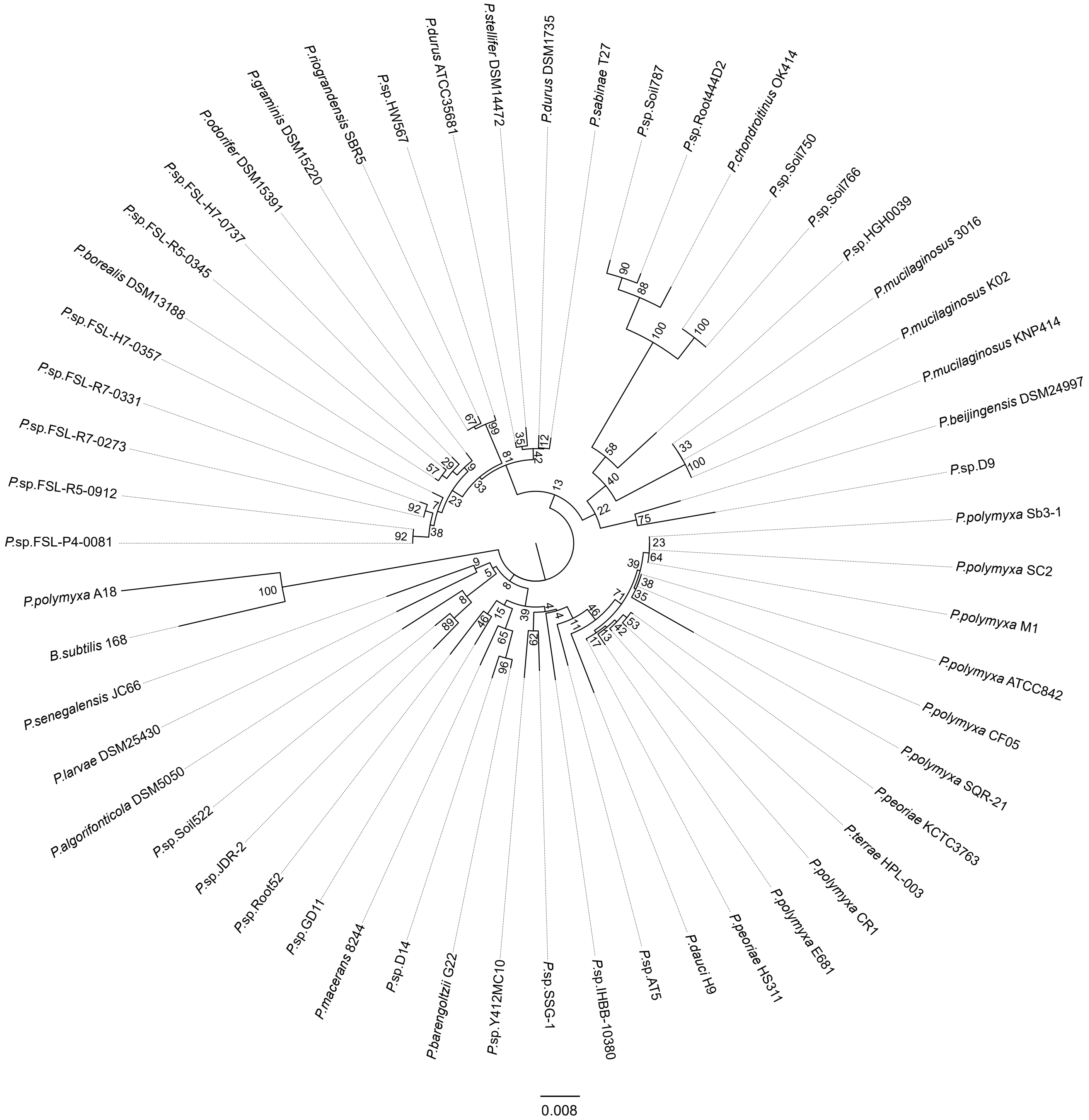

Figure S3. The 16S tree of *Paenibacillus* based on the neighbour-joining method. The numbers in each node denote bootstrap values (maximum 100).

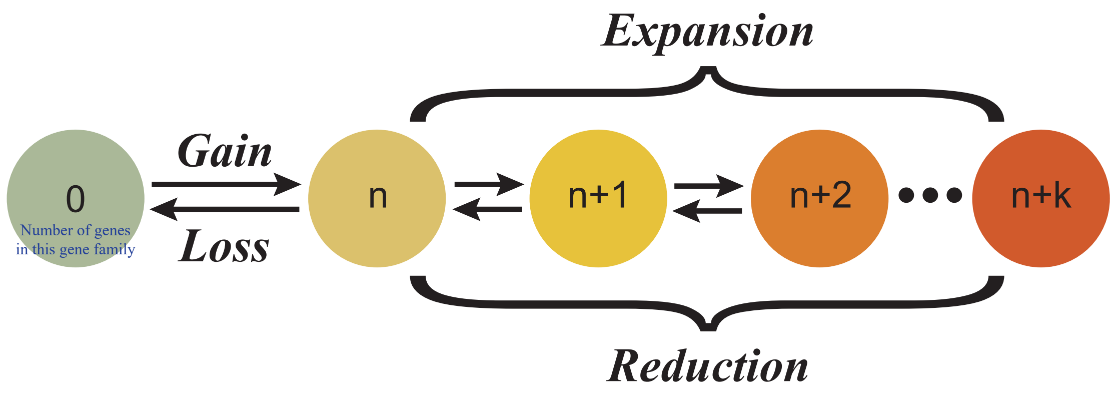

Figure S4. Schematic representation of gain, lose, expansion and reduction.
